# Hydroxyurea utilization among individuals with sickle cell disease in Tennessee: a pooled analysis of claims data

**DOI:** 10.3389/fphar.2025.1693126

**Published:** 2025-12-19

**Authors:** Ayesha Mukhopadhyay, Matthew P. Smeltzer, Judy Dudley, Sherif M. Badawy, Joacy G. Mathias, Allison P. Plaxco, Meredith A. Ray, Andrew D. Wiese, Walter I. Stevens, James G. Gurney, Chinonyelum Nwosu, Jerlym S. Porter, Lisa M. Klesges, Jane S. Hankins, William O. Cooper

**Affiliations:** 1 Division of Epidemiology, Biostatistics, and Environmental Health, School of Public Health, The University of Memphis, Memphis, TN, United States; 2 Department of Health Policy, Vanderbilt University Medical Center, Nashville, TN, United States; 3 Division of Hematology, Oncology and Stem Cell Transplant, Ann & Robert H. Lurie Children’s Hospital of Chicago, Chicago, IL, United States; 4 Department of Epidemiology, Gillings School of Public Health, University of North Carolina, Chapel Hill, NC, United States; 5 Department of Hematology, St. Jude Children’s Research Hospital, Memphis, TN, United States; 6 Department of Psychology, St. Jude Children’s Research Hospital, Memphis, TN, United States; 7 Department of Surgery, Washington University School of Medicine, StLouis, MO, United States; 8 Departments of Global Pediatric Medicine and Hematology, St. Jude Children’s Research Hospital, Memphis, TN, United States; 9 Center for Patient and Professional Advocacy, Vanderbilt University Medical Center, Nashville, TN, United States

**Keywords:** sickle cell anemia, hydroxycarbamide, adherence, compliance, hydroxyurea

## Abstract

**Importance:**

Hydroxyurea reduces severe disease among individuals living with sickle cell disease (SCD). These individuals experience high acute care utilization, but the associations between patterns of hydroxyurea utilization and healthcare utilization are not well investigated.

**Objective:**

This study aimed to determine the association between hydroxyurea use and healthcare utilization among individuals with SCD in Tennessee (TN).

**Design:**

We conducted a population-based, retrospective cohort study of individuals with SCD using secondary data analysis of Tennessee Medicaid, Medicare, and BlueCross BlueShield of Tennessee (BCBS-TN).

**Participants:**

A total of 4,901 individuals with SCD were included in the study.

**Exposure:**

Hydroxyurea adherence was estimated using the medication possession ratio (MPR).

**Main Outcomes and measures:**

The incidence rate ratios of hospitalizations, emergency department visits, and mortality were calculated using negative-binomial models.

**Results:**

The prevalence of hydroxyurea prescription dispensation for the state was low (21% for TennCare, 21% for Medicare, and 17% for BCBS-TN). In TennCare and BCBS-TN, those younger than 18 had more hydroxyurea utilization, and individuals with HbSS or HbSβ^0^ thalassemia filled more hydroxyurea than those with other subtypes (30.5% in TennCare and 23.4% in BCBS-TN). The MPR for the entire state was 19.7%. There was a dose–response relationship between hydroxyurea adherence and the incidence of acute healthcare utilization, except in 18–25-year-olds. We also found lower mortality in those with higher hydroxyurea adherence.

**Conclusion and relevance:**

In our pooled statewide analysis, hydroxyurea MPR was low. Higher hydroxyurea use was associated with decreased acute healthcare utilization and lower mortality. Interventions to support patient adherence and provider prescribing are required to improve health outcomes among individuals with SCD.

## Highlights

1. The prevalence of hydroxyurea prescription dispensation and medication adherence in individuals with sickle cell disease living in Tennessee was low.
2. There was a dose–response relationship between hydroxyurea adherence (calculated by the medication possession ratio) and acute healthcare utilization, except for 18–25-year-olds.
3. Individuals with sickle cell disease who had higher hydroxyurea adherence had lower mortality rates.

## Introduction

In the United States, health-related outcomes for individuals with sickle cell disease (SCD) have improved substantially with increased access to and adherence to evidence-based care in recent years (Neumayr et al., 2019). Along with shortened overall life expectancy, individuals living with SCD have significant impairment in the quality of life (QOL) compared to those without SCD (Stokoe et al., 2022; Freitas et al., 2018). The impact of SCD on QOL is substantial and is associated with numerous and compounding clinical complications leading to increased acute healthcare utilization (Moody, 2022). Individuals with SCD live approximately 2 decades less than those without the disease, with life expectancy in the United States estimated to be 54 years (Lubeck et al., 2019; Lee et al., 2019). Chronic clinical complications of SCD have a profound impact on patients, their caregivers, and society (Asnani et al., 2016; Hood et al., 2021).

Hydroxyurea is associated with decreased SCD-related severe clinical outcomes (e.g., pain crises, stroke, and organ damage) and healthcare resource utilization (Candrilli et al., 2011; Alina et al., 2001; Hankins et al., 2014; Steinberg et al., 2010; Agrawal et al., 2014; Mathias et al., 2020). However, the effectiveness of hydroxyurea is contingent on the initiation of and continued adherence to hydroxyurea (Creary et al., 2015; Ware et al., 2019). Despite overwhelming evidence of safety, efficacy, ease of use, and national guideline recommendations, hydroxyurea remains underutilized, with suboptimal adherence (Candrilli et al., 2011; Badawy et al., 2017a; Dean et al., 2010; Loiselle et al., 2016; Badawy et al., 2017b; Badawy et al., 2017c; Tshilolo et al., 2019; Ware et al., 2016; Wong et al., 2014).

Population-level studies on hydroxyurea utilization are limited, with few studies examining medical claims data from the United States, and those that did included only individuals with continuous Medicaid enrollment (Candrilli et al., 2011; Brousseau et al., 2019; Kang and Barner, 2020; Crego et al., 2020). The extent of the consequences of hydroxyurea underutilization is, thus, understudied at the population level and across different health insurance coverages. To better represent the broad population of people with SCD, we analyzed enrollees of Tennessee Medicaid or TennCare, Medicare, and BlueCross BlueShield of Tennessee (BCBS-TN) with a diagnosis of SCD in a single state. Collectively, these three plans (two governmental plans, TennCare and Medicare; one private plan, BCBS-TN) account for over 80% of Tennesseans with SCD; therefore, our analysis constitutes a statewide, population-based, cross-sectional sample, which is likely representative of the state’s SCD population.

We aimed to investigate the affect of hydroxyurea adherence on acute healthcare utilization and mortality among individuals with SCD in Tennessee. We hypothesized that higher hydroxyurea possession, as a surrogate for medication adherence, was associated with lower acute healthcare utilization and mortality in individuals eligible for hydroxyurea therapy.

## Methods

### Study cohort

We constructed the cohort retrospectively from TennCare, Medicare, and BCBS-TN claims of individuals with SCD during the study period of 1 January 2010 to 30 September 2015. The timeframe was chosen to restrict the analysis to only the ICD-9 classification since the ICD-10 diagnosis codes began being used in October 2015.

TennCare is the state of Tennessee’s Medicaid program. It provides healthcare coverage to people who meet certain income and resource eligibility criteria. More than 1.4 million Tennesseans have access to TennCare, including low-income individuals, pregnant women, children, caretakers of young children and older adults, and adults with disabilities (TennCare, 2025). The Tennessee Medicare program, a federal medical insurance plan, provides coverage to more than 800,000 Tennesseans who are either older than 65 years of age or have a qualifying disability (Disability TCoA, 2025). TennCare currently provides data to the Tennessee Sickle Cell Data Collection Program (SCDC-TN). BCBS-TN is a private, non-profit insurance company that covers 3.3 million members. They are the state’s largest insurance provider, and over 10,000 employers use BCBS-TN to provide health plans for their employees (Tennessee BBo, 2025). Collectively, these three insurance plans cover 83.4% of Tennesseans.

The study cohort constituted participants who satisfied the following criteria: i) had a diagnosis of SCD, defined as having one of the ICD-9 (282.60–64, 68, 69) diagnosis codes; ii) had at least one hospitalization or two outpatient visits or emergency department (ED) encounters (or a combination of one ED visit and one outpatient visit) with the aforementioned diagnoses codes; and iii) were enrolled in TennCare, Medicare, or BCBS-TN plans during the study period. We defined the eligibility for hydroxyurea prescription similarly to the published national guidelines, which are detailed in Supplemental Methods (Yawn et al., 2014). Although the national hydroxyurea guidelines were not published until 2014, we based this decision on evidence from phase-III clinical trials (Charache et al., 1995; Wang et al., 2011) that used similar hydroxyurea initiation criteria to reflect the current indications as a benchmark for future comparisons. The study follow-up time for an individual began on the date of hydroxyurea eligibility fulfillment. The participants’ follow-up period ended at death, loss of enrollment in the health plans, or at the end of the study period, whichever occurred first. Individuals included in the analysis were able to re-enter the study cohort after a lapse in coverage. In this case, the new cohort entrance is considered a new “segment,” which is treated as a new independent observation from the prior study segment.

With approval from the Tennessee Department of Health and the Vanderbilt University Medical Center Institutional Review Boards, we obtained research identification files that consisted of individual-level protected health information, including enrollment records, pharmacy files, and inpatient and outpatient records that were customizable for a specific cohort, such as SCD.

#### Hydroxyurea exposure

Participants were classified according to their estimated amount of exposure to hydroxyurea. We defined current hydroxyurea users as those who had a hydroxyurea prescription dispensed on the day hydroxyurea eligibility was fulfilled. Prescriptions that were not dispensed are not captured in these data sources. The medication possession ratio (MPR) was used as a proxy of hydroxyurea adherence and calculated by dividing the number of days of hydroxyurea prescriptions dispensed by the total number of follow-up days for each patient (Supplemental Methods) (Candrilli et al., 2011; Kang and Barner, 2020; Shah et al., 2019). The hydroxyurea exposure variable was categorized as <40% (reference), 40%–80%, and >80% for TennCare and BCBS-TN and 0%, 0%–20%, and >20% for Medicare to account for small (n < 11) cell sizes. For the negative binomial modeling described in the *Statistical Analysis* subsection below, <40% was used as the reference, but this did not include those who had no utilization. No utilization was defined as a separate category for modeling purposes.

### Outcomes

Study outcomes included acute healthcare utilization (hospitalizations, transfusions, and ED visits) and all-cause mortality. For each outcome, the incidence rate was calculated by enumerating specific utilization (i.e., hospitalizations, transfusions, ED visits, or mortality) occurring in an interval (a time period representing no changes in comorbidities, hydroxyurea eligibility, or hydroxyurea use status) and dividing it by the follow-up time in days during that interval.

#### Covariates

Demographic covariates that were evaluated as potential confounders included the age group (<18 for TennCare and BCBS-TN, 18–25 years, and >25 years), sex, disease type (HbSS or HbSβ^0^ thalassemia versus HbSC, HbSβ^+^ thalassemia, and others), and the region of the state (East, Middle, and West TN). Clinical covariates included the presence of any comorbidities (asthma, diabetes, pulmonary hypertension, stroke, chronic renal failure, congestive heart failure, seizure, hypertension, cancer, hematologic disease other than sickle cell, neuromuscular disease, chromosomal anomaly, cardiovascular and non-cardiovascular congenital anomalies, gastrointestinal disease, HIV or other serious infections, immune deficiency, cardiorespiratory disease, organ transplantation, and other serious ailments) at baseline and a visit to a hematologist in the year prior to study initiation.

### Statistical analyses

Chi-squared and Kruskal–Wallis tests were used to assess whether the prevalence of hydroxyurea use varied by demographic characteristics and whether ordered categories of hydroxyurea adherence varied by demographic characteristics, respectively. A similar analysis was repeated for the subset of individuals with HbSS or HbSβ^0^ thalassemia. For mortality analysis, we reported deaths by categories of hydroxyurea adherence among hydroxyurea-eligible patients who were current users. This could only be reported in TennCare recipients.

Negative binomial models were developed to assess the association between hydroxyurea adherence (exposure) and the number of acute care events, including hospital admissions and ED visits, which were de-duplicated for hospital visits that began in the ED (outcome). In each segment, the amount of time that each person was eligible for hydroxyurea was identified for those under the age of 18 (for TennCare and BCBS-TN), between the ages of 18 and 25, and over the age of 25. Acute care events occurring in the span of hydroxyurea eligible time were counted for each applicable age category within each study segment.

Crude and adjusted negative binomial models were constructed, offset by the amount of hydroxyurea-eligible time that each person contributed to each age category within each study segment. During the model-building process, potential interaction terms were assessed via preliminary modeling, and potential confounders were assessed using the 10% rule (when the difference between the crude and adjusted measures of association was greater than 10%, we concluded that there was confounding). Results were reported by age and by the hydroxyurea adherence levels. Each model’s reference hydroxyurea adherence group was less than 40% hydroxyurea adherence but more than 0. Model-based incidence rate ratios (IRRs) were reported with 95% confidence intervals (CIs). All analyses were performed using the Statistical Analysis System (SAS) software package, version 9.4 (SAS Institute Inc., Cary, NC, United States).

## Results

### Cohort characteristics

**FIGURE 1:**
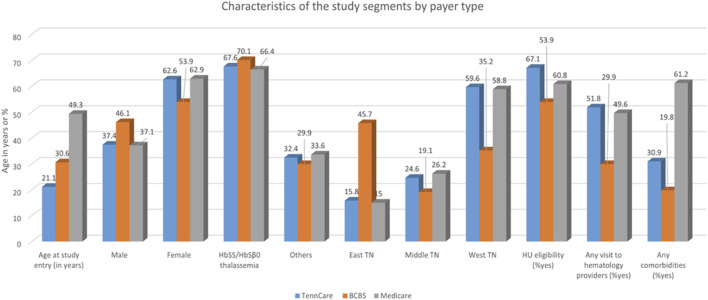
Characteristics of the study segments by payer type.

A total of 4,901 individuals with SCD across three health plans (TennCare, Medicare, and BCBS-TN) were included. There were 3,602 qualifying enrollment segments in TennCare, 774 in Medicare, and 525 in BCBS-TN (Table 1; Figure 1). The age at the study entry was lower for individuals in TennCare than for those in Medicare and BCBS-TN (Table 1; Figure 1). There were more female individuals in all three health plans (63% in TennCare, 63% in Medicare, and 54% in BCBS-TN) (Table 1; Figure 1). Most SCD patients had HbSS or HbSβ^0^ thalassemia (68% in TennCare, 66% in Medicare, and 70% in BCBS-TN) (Table 1; Figure 1). The majority of those covered by governmental plans lived in West TN, while those with private coverage lived in East TN (Table 1; Figure 1). For all three plans, more than half of the patients met the eligibility criteria for hydroxyurea prescription (67% TennCare, 61% Medicare, and 54% BCBS-TN) (Table 1; Figure 1). A greater percentage of individuals with SCD covered by TennCare (52%) visited hematologists before the start of the study period, while most of those covered by Medicare and BCBS-TN did not (Table 1; Figure 1).

**TABLE 1:** Characteristics of the study segments by the payer type.

	TennCare N = 3,602		BCBS-TN (Medicaid removed) N = 525		Medicare N = 774		Aggregate N = 4,901	
Sex	N (%)		N (%)		N (%)		N (%)	
Male	1,346 (37.4)		242 (46.1)		287 (37.1)		1,875 (38.3)	
Female	2,256 (62.6)		283 (53.9)		487 (62.9)		3,026 (61.7)	
SCD phenotype								
HbSS/HbSβ^0^ thalassemia	2,435 (67.6)		368 (70.1)		514 (66.4)		3,317 (67.7)	
Others	1,167 (32.4)		157 (29.9)		260 (33.6)		1,584 (32.3)	
Region (at baseline)								
East	568 (15.8)		240 (45.7)		116 (15.0)		924 (18.8)	
Middle	885 (24.6)		100 (19.1)		203 (26.2)		1,188 (24.2)	
West	2,139 (59.6)		185 (35.2)		455 (58.8)		2,779 (56.7)	
Hydroxyurea eligibility								
Yes (ever eligible)	2,417 (67.1)		283 (53.9)		471 (60.8)		3,171 (64.7)	
No	1,185 (32.9)		242 (46.1)		303 (39.2)		1,730 (35.3)	
Prior visit to hematologist provider within 1 year								
Yes	1,867 (51.8)		157 (29.9)		384 (49.6)		2,408 (49.1)	
No	1,735 (48.2)		368 (70.1)		390 (50.4)		2,493 (50.9)	
Any comorbidities^a^								
Yes	1,113 (30.9)		104 (19.8)		474 (61.2)		1,691 (34.5)	
No	2,489 (69.1)		421 (80.2)		300 (38.8)		3,210 (65.5)	
	Mean (SD)	Median	Mean (SD)	Median	Mean (SD)	Median	Mean (SD)	Median
Age at study entry	21.1 (15.8)	20.0	30.6 (21.2)	28.0	49.3 (19.4)	N/A	N/A	N/A
Number of segments per individual	1.16 (0.41)	1.00	1.04 (0.23)	1.00	1.12 (0.36)	N/A	N/A	N/A
Follow up time (average years per person)	3.50 (2.09)	3.49	2.72 (1.92)	2.19	2.88 (1.98)	N/A	N/A	N/A
Total patient years of follow-up for the cohort	12,590.76	--	1,426.57	--	2,225.47			

^a^ Comorbidities included asthma, diabetes, pulmonary hypertension, stroke, chronic renal failure, congestive heart failure, seizure, hypertension, cancer, hematological diseases other than sickle cell, neuromuscular disease, chromosomal anomaly, both cardiovascular and non-cardiovascular congenital anomalies, gastrointestinal disease, HIV or other serious infections, immune deficiency, cardiorespiratory disease, organ transplantation, and other serious ailments such as coma, cachexia, or vegetative state.

N/A, data not available.

### Prevalence of hydroxyurea utilization

The prevalence estimates of hydroxyurea prescription dispensation for the full study cohorts were low: 21% for TennCare, 21% for Medicare, and 17% for BCBS-TN (Table 2). Variability was present in hydroxyurea utilization across age groups, disease subtypes, regions of residence, prior visits to a hematologist, and health plans. In each study cohort, male individuals were more likely than female individuals to have a hydroxyurea prescription dispensed in TennCare (28.6% vs. 16.8%, *p*-value <0.0001), Medicare (25.1% vs. 18.3%, *p*-value 0.0241), and BCBS-TN (17.4% vs. 16.6%, *p*-value 0.8200) (Table 2). Those aged younger than 18 had more hydroxyurea utilization in TennCare (29% < 18 years vs. 22% 18–25 years vs. 11% > 25 years; p-value < 18 years vs. 16% 18–25 years vs. 7% > 25 years; *p*-value <0.0001) (Table 2). Individuals with HbSS or HbSβ^0^ thalassemia had significantly more hydroxyurea prescriptions dispensed than those without these subtypes (30.5% vs. 1.7%, *p*-value <0.0001 in TennCare, and 23.4% vs. 1.9%, *p*-value <0.0001 in BCBS-TN) (Table 2). Hydroxyurea prescription filling was the highest among those covered under governmental plans in Middle TN (23% in TennCare) and West TN (22% in Medicare) (Table 2).

**TABLE 2:** Prevalence of dispensed hydroxyurea (current hydroxyurea use) among different covariates.

	TennCare N (%)			BCBS-TN N (%)			Medicare N (%)		
	Prescribed hydroxyurea		*p*-value	Prescribed hydroxyurea		*p*-value	Prescribed hydroxyurea		*p*-value
	Yes	No		Yes	No		Yes	No	
	763 (21.3)	2,839 (78.8)		89 (17.0)	436 (83.0)		161 (21.0)	613 (79.2)	
Sex			<0.0001*			0.8200			0.0241*
Male	385 (28.6)	961 (71.4)		42 (17.4)	200 (82.6)		72 (25.1)	215 (74.9)	
Female	378 (16.8)	1,878 (83.2)		47 (16.6)	236 (83.4)		89 (18.3)	398 (81.7)	
Age									
<18 years	460 (29.2)	1,113 (70.8)	<0.0001*	57 (32.7)	117 (67.2)	<0.0001*	N/A	N/A	
18–25 years	155 (21.5)	565 (78.5)		11 (16.4)	56 (83.5)		43 (49.4)	44 (50.6)	<0.0001*
>25 years	148 (11.3)	1,161 (88.7)		21 (7.4)	263 (92.6)		118 (17.2)	569 (82.8)	
SCD phenotype			<0.0001*			<0.0001*			x
HbSS/HbSβ^0^ thalassemia	743 (30.5)	1,692 (69.5)		86 (23.4)	282 (76.6)		x	x	
Others	20 (1.7)	1,147 (98.3)		3 (1.9)	154 (98.1)		x	x	
Region (at baseline)			0.0155*			0.3379			0.6278
East	95 (16.7)	473 (83.3)		44 (18.3)	196 (81.7)		22 (19.0)	94 (81.0)	
Middle	202 (22.8)	683 (77.2)		12 (12.0)	88 (88.0)		39 (19.2)	164 (80.8)	
West	460 (21.5)	1,679 (78.5)		33 (17.8)	152 (82.2)		100 (22.0)	355 (78.0)	
Hydroxyurea eligibility			<0.0001*			<0.0001*			<0.0001*
Yes	710 (29.4)	1,707 (70.6)		73 (25.8)	210 (74.2)		131 (27.8)	340 (72.2)	
No	53 (4.5)	1,132 (95.5)		16 (6.6)	226 (93.4)		30 (9.9)	273 (90.1)	
Prior visit to hematologist provider within 1 year			<0.0001*			0.9221			<0.0001*
Yes	688 (36.8)	1,179 (63.2)		27 (17.2)	130 (82.8)		138 (35.9)	246 (64.1)	
No	75 (4.3)	1,660 (95.7)		62 (16.8)	306 (83.2)		23 (5.9)	367 (94.1)	
Any comorbidities^a^			0.0446*			0.1766			<0.0001*
Yes	Yes HU 213 (19.1)	No HU 900 (80.9)		13 (12.5)	91 (87.5)		74 (15.6)	400 (84.4)	
No	Yes HU 550 (22.1)	No HU 1939 (77.9)		76 (18.0)	345 (82.0)		87 (29.0)	213 (71.0)	
	Mean (SD)	Mean (SD)		Mean (SD)	Mean (SD)		Mean (SD)	Mean (SD)	
Age at study entry	15.4 (12.3)	22.6 (16.3)	<0.0001*	17.0 (12.7)	33.4 (21.6)	<0.0001*	34.7 (11.8)	53.2 (19.1)	
Number of segments	1.09 (0.31)	1.18 (0.43)	<0.0001*	1.03 (0.18)	1.04 (0.24)	0.9442	1.10 (0.3)	1.13 (0.4)	
Follow up time (average years per person)	4.41 (1.8)	3.25 (2.1)	<0.0001*	2.49 (1.7)	2.76 (2.0)	0.5170	3.1 (1.9)	2.8 (2.0)	
Total patient years of follow-up for the cohort	3,362.7	9,228.1	--	221.91	1,204.67	--	507.84	1,717.63	

N/A, data not available.

X, data suppressed to maintain confidentiality.

^a^ Comorbidities included asthma, diabetes, pulmonary hypertension, stroke, chronic renal failure, congestive heart failure, seizure, hypertension, cancer, hematological diseases other than sickle cell, neuromuscular disease, chromosomal anomaly, both cardiovascular and non-cardiovascular congenital anomalies, gastrointestinal disease, HIV or other serious infections, immune deficiency, cardiorespiratory disease, organ transplantation, and other serious ailments such as coma, cachexia, or vegetative state.

HU, hydroxyurea.

Multivariable models were constructed for each individual health plan. For the multivariable models of hydroxyurea use, female individuals were less likely than male individuals to have a hydroxyurea prescription dispensed under governmental plans (TennCare and Medicare) (OR: 0.872, 95% CI: (0.75, 1.01) for TennCare and OR: 0.776, 95% CI: (0.51, 1.18) for Medicare) (Supplementary Table S1). Those with HbSS or HbSβ^0^ thalassemia subtypes had higher odds of hydroxyurea use (OR: 1.752, 95% CI: (1.51, 2.03); OR: 2.03, 95% CI: (1.36, 3.03); OR: 1.852, 95% CI: (1.24, 2.76) in TennCare, Medicare, and BCBS-TN, respectively) (Supplementary Table S1). In TennCare, the odds of being dispensed hydroxyurea were lower among those living in East TN (OR: 0.65, 95% CI: 0.53, 0.79) than among those in West TN (Supplementary Table S1). Hydroxyurea eligibility was associated with having a hydroxyurea prescription dispensed. Among TennCare enrollees, those who were not eligible for hydroxyurea had an odds ratio of 0.34 with 95% CI (0.29, 0.40); Medicare enrollees had an odds ratio of 0.04 with 95% CI (0.02, 0.09), and BCBS-TN enrollees had an odds ratio of 0.40 with 95% CI (0.27, 0.59) of prescribed hydroxyurea (Supplementary Table S1). For all three plans, those with comorbidities had higher odds of ever using hydroxyurea (Supplementary Table S1).

### Hydroxyurea adherence among patients who used hydroxyurea

The hydroxyurea adherence or MPR for the entire state of TN among those who ever had a prescription dispensed was 19.7%. Variability in MPR was present across age groups, disease subtypes, regions, prior visits to a hematologist, and health plans. For those with low adherence (<40%) in TennCare, more individuals had disease subtypes other than HbSS or HbSβ^0^ thalassemia (99% vs. 85%) (Supplementary Table S2). Those with low adherence (<40%) had a higher prevalence of no prior hematologist visits (98% vs. 82%) (Supplementary Table S2). A greater percentage of those with low adherence had other comorbidities (91% vs. 88%) (Supplementary Table S2). We obtained similar results for those with BCBS-TN and Medicare coverage (Supplementary Tables S3, S4).

### Association between hydroxyurea adherence and acute healthcare utilization

**FIGURE 2:**
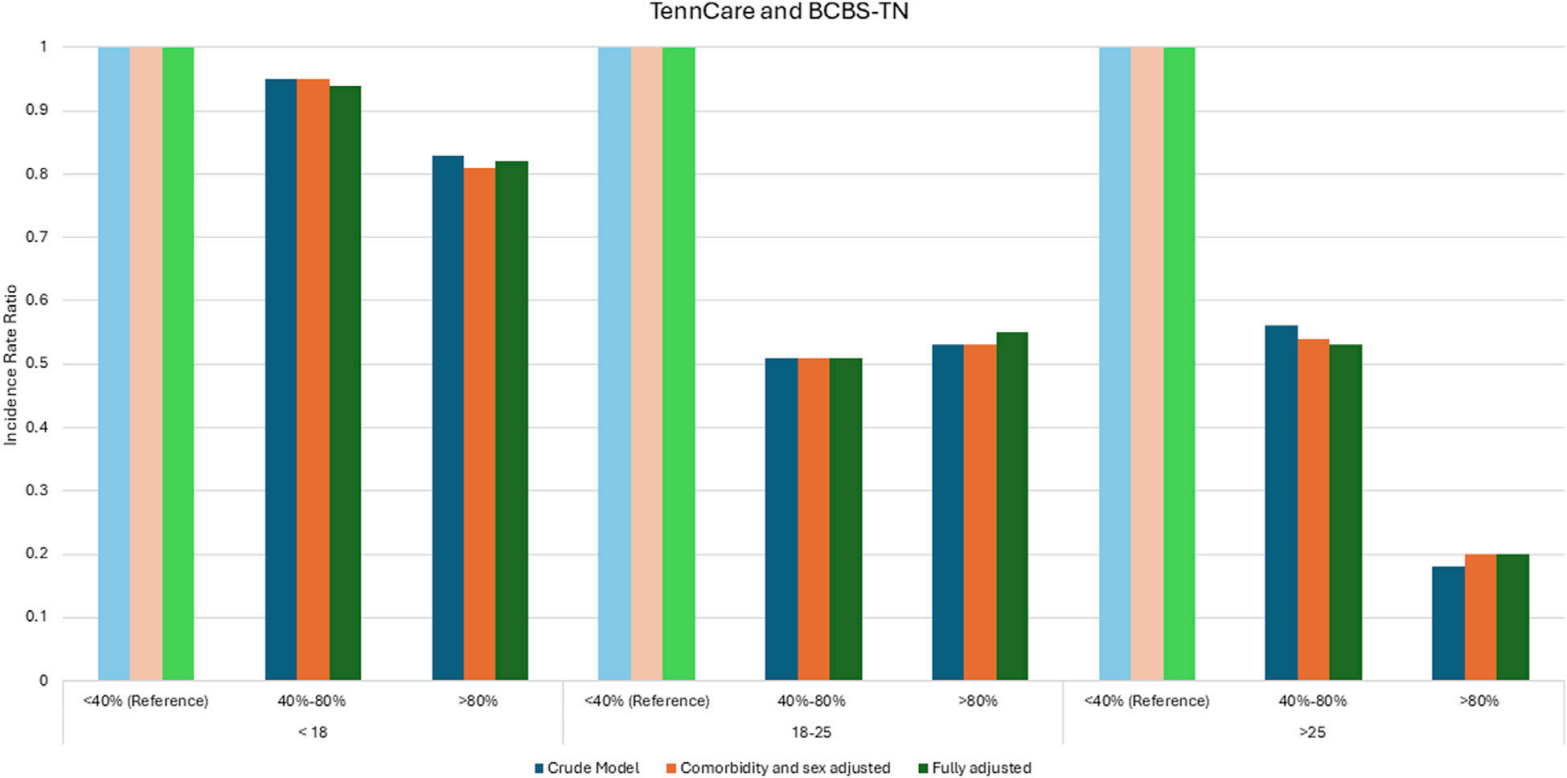
Incidence rate ratios between hydroxyurea adherence and the incidence of acute care utilization.

Our analysis demonstrated an association between hydroxyurea adherence and the incidence of acute care utilization for TennCare and BCBS-TN enrollees. In fully adjusted models, those with >80% MPR had lower incidence of acute care utilization than those with <40% MPR in individuals <18 years [IRR: 0.82 (95% CI: 0.63, 1.07)], 18–25 years [IRR: 0.55 (95% CI: 0.37, 0.81)], and those >25 years [IRR: 0.2 (95% CI: 0.1, 0.41)]; those with 40%–80% MPR had lower incidence of acute care utilization than those with <40% MPR in individuals <18 years [IRR: 0.94 (95% CI: 0.72, 1.23)], 18–25 years [IRR: 0.51 (95% CI: 0.33, 0.78)], and those >25 years [IRR: 0.53 (95% CI: 0.35, 0.79)] (Figure 2). IRRs were statistically significant at the 0.05 level, except in the <18-year-old group. The IRR of the crude models, comorbidity and sex-adjusted models, and fully adjusted models demonstrate a dose response relationship, such that higher MPR values were associated with lower acute care utilization (ED and hospital admissions), except in 18–25-year-olds (Figure 2).

### Hydroxyurea adherence and healthcare utilization variability by sickle genotypes

The previous analyses were repeated for the 2,597 members of the cohort with HbSS or HbSβ^0^ thalassemia subtypes. Multivariable models were constructed for each individual health plan. For the multivariable models of hydroxyurea adherence, female individuals were less likely to have been prescribed hydroxyurea under TennCare (OR: 0.88, 95% CI: 0.73, 1.05) but were more likely under Medicare (OR: 1.12, 95% CI: 0.65, 1.93) and BCBS-TN (OR: 1.50, 95% CI: 0.93, 2.40) (Supplementary Table S5). The odds of being dispensed hydroxyurea were lower among those living in East TN (OR: 0.74, 95% CI: (0.57, 0.96); and OR: 0.97, 95% CI: (0.57, 1.64) for TennCare and BCBS-TN, respectively) and Middle TN (OR: 0.96, 95% CI: (0.78, 1.19); and OR: 0.76, 95% CI: (0.38, 1.53) for TennCare and BCBS-TN, respectively) (Supplementary Table S5). Hydroxyurea eligibility was associated with adherence. Among TennCare enrollees, those who were not eligible had an odds ratio of 0.29 with 95% CI (0.24, 0.35), Medicare enrollees had an odds ratio of 0.06 with 95% CI (0.03, 0.14), and BCBS-TN enrollees had an odds ratio of 0.58 with 95% CI (0.36, 0.94) (Supplementary Table S5). For all three plans, those with comorbidities had higher odds of hydroxyurea adherence (Supplementary Table S5).

### Hydroxyurea adherence and mortality

For hydroxyurea-eligible patients with TennCare coverage, there were 122 (5.9%) deaths in those who had <40% hydroxyurea adherence (Supplementary Table S6). Those with 40%–80% and >80% adherence had lower mortality (4% and 1.6%, respectively) (Supplementary Table S6). Death data were unavailable for those with BCBS-TN coverage (Supplementary Table S7). We were unable to report adverse events, including deaths, for Medicare recipients who were hydroxyurea-eligible because of small cell sizes. Even though the numbers are low, we found that adherence to hydroxyurea treatment was associated with lower mortality.

## Discussion

People with SCD face substantial disease complications, shorter life expectancy, and increased healthcare utilization (Yang et al., 2022). Despite the existing evidence of the safety and efficacy of hydroxyurea, it remains vastly underutilized. Our study described hydroxyurea use and adherence patterns and their association with acute healthcare utilization and mortality in the most comprehensive claims data analysis for the state of TN. We found that hydroxyurea dispensation and adherence in the state were low. Those younger than 18 had higher hydroxyurea utilization, and male individuals filled more hydroxyurea prescriptions. Those who had HbSS or HbSβ^0^ thalassemia also filled more hydroxyurea prescriptions than other subtypes. The likelihood of filling a hydroxyurea prescription and adhering to the medication was higher among individuals who visited a hematologist and in those who were eligible to receive hydroxyurea. Hydroxyurea adherence was inversely associated with acute healthcare utilization and deaths. Higher adherence to hydroxyurea was associated with fewer ED visits and hospitalizations and lower mortality.

Overall, <50% of those with SCD in our state had hydroxyurea adherence, which is similar to the reports of previously published studies using claims datasets (Candrilli et al., 2011; Shah et al., 2020). Clinical complications secondary to underutilization and low adherence to hydroxyurea adversely affect QOL in those with SCD (Nwenyi et al., 2014). Previous studies have demonstrated that prescription possession and adherence to hydroxyurea were associated with decreased frequency of pain crises, reduced healthcare utilization, and, thus, improved QOL (Ballas et al., 2006). Some barriers to adherence include the fear of potential side effects, misunderstanding the need for the medication, lack of access to specialized care, and reduced patient involvement in informed decision-making about disease processes and treatment options (Badawy et al., 2017a; Loiselle et al., 2016; Badawy et al., 2017b; Badawy et al., 2017c; Sobota et al., 2011; Badawy et al., 2018a; Badawy et al., 2018b; Badawy et al., 2016; Badawy et al., 2018c; Alberts et al., 2020). Newer techniques, such as telehealth or mobile health apps, need to be further evaluated for their potential to enhance medication adherence (Hankins et al., 2023; Jacob et al., 2021).

We found that those who visited a hematologist had a higher likelihood of being prescribed hydroxyurea and having higher medication adherence. In the era of our study, treatment with hydroxyurea was well-supported by high-quality clinical trials, but no consensus guidelines were available until 2014. Follow-up studies should evaluate whether the publication of consensus guidelines led to increased hydroxyurea prescriptions in individuals who had not previously visited a hematologist. It is well-documented that the lack of detailed knowledge (Sobota et al., 2011; Travis et al., 2020; Lunyera et al., 2017; Kanter et al., 2020) many providers have about the treatment of hemoglobinopathies such as SCD and negative perceptions toward patients with SCD are critical barriers to hydroxyurea use (Pizzo et al., 2023; Smeltzer et al., 2021). Non-adherence is further exacerbated by patients’ ongoing mistrust of healthcare systems, pharmaceutical companies, and physicians’ prescribing practices (Matthie et al., 2016; Mathur et al., 2016; Renedo et al., 2019). These barriers must be addressed to reduce suboptimal hydroxyurea use (Varty and Popejoy, 2020; Smaldone et al., 2018).

In this cohort, we found that those with the SCD subtypes HbSS or HbSβ^0^ thalassemia had more hydroxyurea prescriptions and adherence. This may be explained by the clinical trial eligibility inclusion criteria and recommendations for SCD that have specific criteria for initiating hydroxyurea for certain SCD subtypes and less clear recommendations for other genotypes (National Heart, 2014). We also did not observe a dose–response relationship between hydroxyurea adherence and the incidence of acute care utilization in young adults (18–25-year-olds). Young adulthood is the peak period of acute utilization in those with SCD. Young adults experience changes in care during the pediatric-to-adult transition, a higher potential for disrupted follow-up, and other life transitions, such as relocation, that might impact this association or our ability to evaluate it in this type of analysis. It is also possible that hydroxyurea alone is not sufficient to reduce acute utilization in this age group, and combination therapy with newer agents and structured adult care transition services is needed.

Our study had several limitations. First, we analyzed individuals covered by TennCare, Medicare, and BCBS-TN. Although this cohort includes a large number of individuals with SCD in TN, we could not analyze those who were uninsured or covered by commercial insurance other than BCBS-TN. The true proportion of individuals with SCD who use hydroxyurea could be greater than our estimate if those with commercial insurance other than BCBS-TN had higher utilization. Conversely, those who are uninsured may utilize hydroxyurea less than we have estimated. Future studies within the statewide surveillance program for SCD in TN, the SCDC-TN (Tennessee Sickle Cell Data Collection Program) surveillance program (funded by the Centers for Disease Control and Prevention), will provide even more comprehensive data in the future (Prevention TCfDCa, 2023). Second, we classified genotypes based on previous definitions, which may under- or overestimate disease burden. Additionally, we did not account for two important genetic modifiers of SCD, the hemoglobin F levels (Bao et al., 2019) and co-inherited alpha thalassemia (Hatzlhofer et al., 2021)—none of which were available in our claims datasets. Third, we used hydroxyurea prescription possession as a surrogate for hydroxyurea adherence, which may introduce misclassification bias due to variations in dosing or daily adherence. Additionally, the cut-off for MPR that we applied to the Medicare population, which was necessary due to sparse data, limits the comparability of this population. Fourth, national hydroxyurea guidelines were not published until 2014; therefore, providers’ decision to prescribe hydroxyurea would have been based on evidence from phase-III clinical trials during the majority of this study. These data may not reflect the more recent eligibility and utilization trends. Our study analysis from 1 January 2010 to 30 September 2015 serves as a benchmark for a planned updated analysis. Finally, the population we analyzed was restricted to only one state, which may not necessarily quantify the countrywide disease burden and hydroxyurea utilization patterns for SCD (Ballas et al., 2018).

## Conclusion

Individuals with SCD in the state of TN have very low hydroxyurea utilization and adherence rates. The suboptimal use of this safe and effective medication increases healthcare utilization. The factors associated with increased hydroxyurea adherence included being younger than 18, visiting a hematologist, having HbSS or HbSβ^0^ thalassemia, and being eligible to receive hydroxyurea. We also found that better adherence to hydroxyurea was associated with lower mortality. To further alleviate the sufferings of those with SCD, factors associated with hydroxyurea utilization and adherence need to be evaluated and addressed.

## Data Availability

The original contributions presented in the study are included in the article/Supplementary Material; further inquiries can be directed to the corresponding authors.

## Abbreviations

SCD, sickle cell disease; ED, emergency department; TN, Tennessee; TennCare, TN Medicaid; MPR, medication possession ratio; IRR, incidence rate ratio.
