# Supplementary material for: Hydroxyurea utilization among individuals with sickle cell disease in Tennessee: a pooled analysis of claims data
Source: Front Pharmacol. 2025 Dec 19;16:1693126. doi: 10.3389/fphar.2025.1693126 (PMC12757755; doi:10.3389/fphar.2025.1693126)
Supplement: Supplementary file 1 [file Supplementaryfile1.docx]

**Supplemental Table 1.** Multivariable models of hydroxyurea use (ever vs. none) among different payer types.

|  | TennCare | | BCBS-TN | | Medicare | |
| --- | --- | --- | --- | --- | --- | --- |
|  | OR (95%CI) | p-value | OR (95%CI) | p-value | OR (95%CI) | p-value |
| **Sex** |  |  |  |  |  |  |
| Male | 1.0 |  | 1.0 |  | 1.0 |  |
| Female | 0.872 (0.75-1.01) | 0.0703 | 1.182 (0.81-1.72) | 0.3824 | 0.776 (0.51-1.18) | 0.2387 |
| **SCD Phenotype** |  |  |  |  |  |  |
| HbSS/HbSβ^0^ thalassemia | 1.752 (1.51-2.03) | < 0.0001* | 1.852 (1.24-2.76) | 0.0024* | 2.030 (1.36-3.03) | 0.0005* |
| Other | 1.0 |  | 1.0 |  |  |  |
| **Region** |  |  |  |  |  |  |
| East | 0.648 (0.53-0.79) | < 0.0001* | 0.981 (0.64-1.50) | 0.9297 | 0.873 (0.51-1.50) | 0.6243 |
| Middle | 0.884 (0.74-1.05) | 0.1578 | 0.962 (0.56-1.64) | 0.8859 | 1.294 (0.79-2.11) | 0.3019 |
| West | 1.0 |  | 1.0 |  | 1.0 |  |
| **Hydroxyurea Eligibility** |  |  |  |  |  |  |
| Yes | 1.0 |  | 1.0 |  |  | 1.0 |
| No | 0.341 (0.29-0.40) | < 0.0001* | 0.401 (0.27-0.59) | <0.0001* | 0.046 (0.02, 0.09) | <0.0001* |
| **Prior visit to Hematology Provider within 1 year** |  |  |  |  |  |  |
| Yes | 1.0 |  | 1.0 |  | 1.0 |  |
| No | 0.356 (0.31-0.41) | < 0.0001* | 0.642 (0.51-0.81) | 0.0003* | 0.557 (0.37-0.84) | 0.0049* |
| **Any Comorbidities^1^** |  |  |  |  |  |  |
| Yes | 1.0 |  | 1.0 |  | 1.0 |  |
| No | 0.475 (0.40-0.56) | < 0.0001* | 0.916 (0.58-1.44) | 0.7037 | 0.547 (0.37-0.81) | 0.0030* |
| **Age - Study Entry**  18-25 years vs <18  >25 years vs < 18  18-25 years vs > 25 for Medicare | 1.17 (0.97-1.42)  1.23 (1.05-1.44) | 0.1073  0.0106* | 1.036 (0.60-1.80)  0.988 (0.75-1.31) | 0.8992  0.9329 | 2.64 (1.04-6.70) | 0.0412* |
| **# of Segments**  Segment 2 vs 1  Segment 3 vs 1  Segment 4 vs 1 | 0.618 (0.50-0.76)  0.439 (0.25-0.78)  1.213 (0.13-11.7) | <0.0001*  0.0048*  0.8674 | 0.406 (0.30-0.56)  0.138 (0.04-0.44)  1.032 (0.11-9.95) | <0.0001*  0.0008*  0.9780 | 0.488 (0.28-0.86  0.960 (0.114-8.059)  N/A | 0.0123*   0.9697 |
| **Follow Up Time**  (2-4 Yrs vs 0-2 Yrs)  (4-5.75 vs 0-2 Yrs) | 2.187 (1.81-2.65)  3.954 (3.96-4.68) | < 0.0001*  < 0.0001* | 1.227 (0.81-1.85)  1.290 (0.87-1.91) | 0.3297  0.2032 | 6.740 (3.50-12.97)  5.289 (3.01-9.30) | <0.0001*  <0.0001* |

^1^Comorbidities included: Asthma, diabetes, pulmonary hypertension, stroke, chronic renal failure, congestive heart failure, seizure, hypertension, cancer, hematologic disease other than sickle cell, neuromuscular disease, chromosomal anomaly, both cardiovascular and non-cardiovascular congenital anomalies, gastrointestinal disease, HIV or other serious infections, immune deficiency, cardio-respiratory disease, organ transplantation, and other serious ailments like coma, cachexia, or vegetative state

N/A Data not available

**Supplemental Table 2.** Proportion of hydroxyurea adherence among TennCare Participants

|  |  |  |  |
| --- | --- | --- | --- |
|  | <40%  N (%) | 40 to 80%  N (%) | >80%  N (%) |
| **Sex** |  |  |  |
| Male | 1144 (85.0) | 99 (7.4) | 103 (7.6) |
| Female | 2082 (92.3) | 90 (4.0) | 84 (3.7) |
| **SCD Phenotype** |  |  |  |
| HbSS/HbSβ^0^ thalassemia | 2074 (85.2) | 181 (7.4) | 180 (7.4) |
| Other | 1152 (98.7) | 8 (0.7) | 7 (0.6) |
| **Region** |  |  |  |
| East | 521 (91.7) | 39 (6.9) | 8 (1.4) |
| Middle | 811 (91.6) | 53 (6.0) | 21 (2.4) |
| West | 1885 (88.1) | 97 (4.5) | 157 (7.4) |
| **Hydroxyurea Eligibility** |  |  |  |
| Yes | 2057 (85.1) | 176 (7.3) | 184 (7.6) |
| No | 1169 (98.6) | 13 (1.1) | 3 (0.3) |
| **Prior Visit to Hematologist Provider within 1 year** |  |  |  |
| Yes | 1531 (82.0) | 168 (9.0) | 168 (9.0) |
| No | 1695 (97.7) | 21 (1.2) | 19 (1.1) |
| **Any Comorbidities^1^** |  |  |  |
| Yes | 1020 (91.6) | 51 (4.6) | 42 (3.8) |
| No | 2206 (88.6) | 138 (5.5) | 145 (5.8) |
|  | Mean (SD) | Mean (SD) | Mean (SD) |
| **Age at Study Entry** | 21.96 (15.96) | 15.16 (14.61) | 11.57 (7.43) |
| **Number of Segments** | 1.16 (0.41) | 1.10 (0.36) | 1.11 (0.34) |
| **Follow Up Time (Avg years per person)** | 3.41 (2.09) | 4.19 (1.92) | 4.28 (2.09) |
| **Total Patient-Years**  **Follow-up** | 10,997.7 | 792.4 | 800.7 |

^1^Comorbidities included: Asthma, diabetes, pulmonary hypertension, stroke, chronic renal failure, congestive heart failure, seizure, hypertension, cancer, hematologic disease other than sickle cell, neuromuscular disease, chromosomal anomaly, both cardiovascular and non-cardiovascular congenital anomalies, gastrointestinal disease, HIV or other serious infections, immune deficiency, cardio-respiratory disease, organ transplantation, and other serious ailments like coma, cachexia, or vegetative state

**Supplemental Table 3**. Proportion of hydroxyurea adherence among BCBS-TN Participants

|  |  |  |  |
| --- | --- | --- | --- |
|  | <40%  N (%) | 40 to 80%  N (%) | >80%  N (%) |
| **Sex** |  |  |  |
| Male | 219 (90.5) | 10 (4.1) | 13 (5.4) |
| Female | 254 (89.8) | 20 (7.1) | 9 (3.2) |
| **SCD Phenotype** |  |  |  |
| HbSS/HbSβ^0^ thalassemia | 318 (86.4) | 29 (7.0) | 21 (5.7) |
| Other | 166 (98.7) | 1 (0.6) | 1 (0.6) |
| **Region** |  |  |  |
| East | 213 (88.8) | 16 (6.7) | 11 (4.6) |
| Middle | 96 (96.0) | 3 (3.0) | 1 (1.0) |
| West | 165 (88.6) | 11 (6.0) | 10 (5.4) |
| **Hydroxyurea Eligibility** |  |  |  |
| Yes | 236 (83.4) | 26 (9.2) | 21 (7.4) |
| No | 237 (97.9) | 4 (1.6) | 1 (0.4) |
| **Any Visit to Hematologist Provider within 1 year** |  |  |  |
| Yes | 145 (92.4) | 7 (4.5) | 5 (3.2) |
| No | 328 (89.1) | 23 (6.2) | 17 (4.6) |
| **Any Comorbidities^1^** |  |  |  |
| Yes | 97 (93.3) | 5 (4.8) | 2 (1.9) |
| No | 376 (89.3) | 25 (5.9) | 20 (4.8) |
|  | Mean (SD) | Mean (SD) | Mean (SD) |
| **Age at Study Entry** | 32.2 (21.4) | 16.8 (12.3) | 15.6 (14.9) |
| **Number of Segments** | 1.10 (0.34) | 1.07 (0.25) | 1.00 (0.0) |
| **Follow Up Time (Avg years per person)** | 2.75 (1.94) | 2.65 (1.91) | 2.16 (1.51) |
| **Total Patient-Years**  **Follow-up** | 1299.3 | 79.61 | 47.62 |

^1^Comorbidities included: Asthma, diabetes, pulmonary hypertension, stroke, chronic renal failure, congestive heart failure, seizure, hypertension, cancer, hematologic disease other than sickle cell, neuromuscular disease, chromosomal anomaly, both cardiovascular and non-cardiovascular congenital anomalies, gastrointestinal disease, HIV or other serious infections, immune deficiency, cardio-respiratory disease, organ transplantation, and other serious ailments like coma, cachexia, or vegetative state

**Supplemental Table 4**. Proportion of hydroxyurea adherence among Medicare Participants

|  |  |  |  |
| --- | --- | --- | --- |
|  | <0%  N (%) | >0 to 20%  N (%) | >20 to 100%  N (%) |
| **Sex** |  |  |  |
| Male | 213 (74.9) | 29 (10.1) | 43 (15.0) |
| Female | 398 (81.7) | 50 (10.3) | 39 (8.0) |
| **SCD Phenotype** |  |  |  |
| HbSS/HbSβ^0^ thalassemia | x | x | x |
| Other | x | x | x |
| **Region** |  |  |  |
| East and Middle | 258 (80.8) | 23 (7.2) | 38 (11.9) |
| West | 355 (78.0) | 56 (12.3) | 44 (9.7) |
| **Hydroxyurea Eligibility** |  |  |  |
| Yes | 340 (72.1) | 67 (14.2) | 64 (13.6) |
| No | 273 (90.1) | 12 (4.0) | 18 (5.9) |
| **Prior visit to Hematologist Provider within 1 year** |  |  |  |
| Yes | 246 (64.1) | 68 (17.7) | 70 (18.2) |
| No | 367 (94.1) | 11 (2.8) | 12 (3.1) |
| **Any Comorbidities^1^** |  |  |  |
| Yes | 400 (84.4) | 38 (8.0) | 36 (7.6) |
| No | 213 (71.0) | 41 (13.7) | 46 (15.3) |
|  | Mean (SD) | Mean (SD) | Mean (SD) |
| **Age at Study Entry** | 53.2 (19.2) | 32.3 (9.6) | 37.1 (13.3) |
| **Number of Segments** | 1.13 (0.38) | 1.06 (0.25) | 1.13 (0.38) |
| **Follow Up Time (Avg years per person)** | 2.80 (1.99) | 3.48 (1.79) | 2.84 (1.99) |
| **Total Patient-Years**  **Follow-up** | 1023.4 | 1272.2 | 1036.3 |

X Data suppressed to maintain confidentiality

^1^Comorbidities included: Asthma, diabetes, pulmonary hypertension, stroke, chronic renal failure, congestive heart failure, seizure, hypertension, cancer, hematologic disease other than sickle cell, neuromuscular disease, chromosomal anomaly, both cardiovascular and non-cardiovascular congenital anomalies, gastrointestinal disease, HIV or other serious infections, immune deficiency, cardio-respiratory disease, organ transplantation, and other serious ailments like coma, cachexia, or vegetative state

**Supplemental Table 5.** Sensitivity Analysis: Reproduction of multivariable analysis restricted to only HbSS/HBSβ^0^ participants.

|  | TennCare (N=2435) | | BCBS-TN (N=368) | | Medicare (N=514) |  |  |  |
| --- | --- | --- | --- | --- | --- | --- | --- | --- |
|  | OR (95%CI) | p-value | OR (95%CI) | p-value | OR (95%CI) | p-value | | |
| **Sex** |  |  |  |  |  |  | | |
| Male | 1.0 |  | 1.0 |  | 1.0 |  | | |
| Female | 0.878 (0.73-1.05) | 0.1638 | 1.496 (0.93-2.40) | 0.0941 | 1.12 (0.65-1.93) | 0.6780 | | |
| **Region** |  |  |  |  |  |  | | |
| East | 0.740 (0.57-0.96) | 0.0221* | 0.969 (0.57-1.64) | 0.0956 | 1.015 (0.45-2.29) | 0.9716 | | |
| Middle | 0.963 (0.78-1.19) | 0.7337 | 0.760 (0.38-1.53) | 0.4399 | 1.438 (0.74-2.79) | 0.2818 | | |
| West | 1.0 |  | 1.0 |  | 1.0 |  | | |
| **Hydroxyurea Eligibility** |  |  |  |  |  |  | | |
| Yes | 1.0 |  | 1.0 |  | 1.0 |  | | |
| No | 0.288 (0.24-0.35) | < 0.0001* | 0.584 (0.36-0.94) | 0.0267* | 0.069 (0.03-0.14) | <0.0001* | | |
| **Prior Visit to Hematologist Provider within 1 year** |  |  |  |  |  |  | | |
| Yes | 1.0 |  | 1.0 |  | 1.0 |  | | |
| No | 0.321 (0.27-0.39) | < 0.0001* | 0.690 (0.41-1.17) | 0.1671 | 0.830 (0.48-1.43) | 0.5051 | | |
| **Any Comorbidities^1^** |  |  |  |  |  | |  |  |
| Yes | 1.0 |  | 1.0 |  | 1.0 |  | | |
| No | 0.418 (0.33-0.52) | <0.0001* | 0.756 (0.39-1.47) | 0.4078 | 0.395 (0.23-0.69) | 0.0011* | | |
| **Age - Study Entry**  18-25 years vs <18  >25 years vs < 18  18-25 years vs >25 for Medicare | 1.131 (0.89-1.44)  1.315 (1.06-1.63) | 0.3191  0.0119* | 1.782 (0.80-3.98)  1.584 (0.96-2.61) | 0.1593  0.0714 | N/A  N/A  1.971 (0.76-5.10) | | N/A  N/A  0.1617 |  |
| **# of Segments**  Segment 2 vs 1  Segment 3 vs 1  Segment 4 vs 1 | 0.582 (0.45-0.76)  0.331 (0.15-0.66)  >999.9 (<0.001->999) | < 0.0001*  0.0073*  0.9739 | 0.508 (0.14-1.84)  <0.001 (<0.001->999)  <0.001 (<0.001->999) | 0.3019  0.9926  0.9926 | 0.538 (0.24-1.17)  >999.9 (<0.001->999)  >999.9 (<0.001->999) | | 0.1195  0.9910  0.9948 |  |
| **Follow Up Time**  (2-4 Yrs vs 0-2 Yrs)  (4-5.75 vs 0-2 Yrs) | 2.113 (1.66-2.70)  4.397 (3.55-5.45) | < 0.0001*  < 0.0001* | 1.584 (0.89-2.83)  1.848 (1.01-3.38) | 0.1193  0.0465* | 6.421 (2.48-16.63)  3.767 (1.84-7.73) | 0.0001*  0.0003* | | |

^1^Comorbidities included: Asthma, diabetes, pulmonary hypertension, stroke, chronic renal failure, congestive heart failure, seizure, hypertension, cancer, hematologic disease other than sickle cell, neuromuscular disease, chromosomal anomaly, both cardiovascular and non-cardiovascular congenital anomalies, gastrointestinal disease, HIV or other serious infections, immune deficiency, cardio-respiratory disease, organ transplantation, and other serious ailments like coma, cachexia, or vegetative state

N/A Data not available

**Supplemental Table 6.** Proportion of hydroxyurea adherence and adverse events among TennCare participants for hydroxyurea eligible patients only (Total number of events = 4831)

|  | Hydroxyurea Adherence | | |
| --- | --- | --- | --- |
|  | <40%  N (%) | >=40 to 80%  N (%) | >80%  N (%) |
| Total | 4032 | 426 | 373 |
| Emergency Dept Visits | 1691 (82.2) | 152 (86.4) | 143 (77.7) |
| Hospitalizations | 1505 (73.2) | 153 (86.9) | 137 (74.5) |
| Transfusions | 714 (34.7) | 114 (64.8) | 90 (48.9) |
| Deaths | 122 (5.9) | 7 (4.0) | 3 (1.6) |

Supplemental Table 7. Proportion of hydroxyurea adherence and adverse events among BCBS-TN participants for hydroxyurea eligible patients only (Total number of events = 496)

|  | Hydroxyurea Adherence | | |
| --- | --- | --- | --- |
|  | <40%  N (%) | >=40 to 80%  N (%) | >80%  N (%) |
| Total | 420 | 45 | 31 |
| Emergency Dept Visits | 155 (65.7) | 17 (65.4) | 14 (66.7) |
| Hospitalizations | 176 (74.6) | 17 (65.4) | 11 (52.4) |
| Transfusions | 89 (37.7) | 11 (42.3) | 6 (28.6) |
| Deaths | N/A | N/A | N/A |

N/A Data not available
