# Supplementary material for: Hydroxyurea utilization among individuals with sickle cell disease in Tennessee: a pooled analysis of claims data
Source: Front Pharmacol. 2025 Dec 19;16:1693126. doi: 10.3389/fphar.2025.1693126 (PMC12757755; doi:10.3389/fphar.2025.1693126)
Supplement: Supplementary file 2 [file Supplementaryfile2.docx]

**Supplemental Methods**

*Hydroxyurea Eligibility Guidelines*

For each day in the study period, persons with sickle cell disease were characterized as to whether they meet the current guidelines for hydroxyurea use, which include:

Children of ages [9 months, <18 years of age)

Adults (>18 years of age) with:

3 or more hospitalizations in the prior 12 months. For the primary analysis, we will include adults qualifying for the cohort who have >3 hospitalizations for any reason within a 12 month period. For sensitivity analyses, we will include only those who have >3 hospitalizations with at least one diagnosis field containing ICD-9 codes for crisis (282.62, 282.42, 282.64, 282.69)

Cohort members who are on hydroxyurea within the first 30 days of follow-up will be considered as meeting the above guidelines.

For children who transition to adulthood and are on hydroxyurea on their 18^th^ birthday, we will consider all subsequent person-time as meeting hydroxyurea guidelines. For children who transition to adulthood and are not on hydroxyurea on their 18^th^ birthday, we will set their qualification for hydroxyurea moving forward as we do for all adults in the cohort. Once adults meet guidelines for hydroxyurea, all subsequent person time will be considered as meeting the guidelines.

**Hydroxyurea Exposure and Medication Possession Ratio (MPR)**

The entire study cohort was classified according to the amount of exposure to hydroxyurea. Hydroxyurea was identified using NDC codes and depending on the availability of the information, patient medical records were used as a supplement to capture hydroxyurea dispensation. We did not have access to information on medication use during hospitalization and therefore hospitalization time was excluded from follow‐up for MPR calculations when applicable. When a study participant had not filled a prescription for hydroxyurea in the preceding year by study start date, we defined them as a “never” hydroxyurea user.
